# The Sensitivity of Magnetic Resonance Imaging and Ultrasonography in Detecting Rotator Cuff Tears

**DOI:** 10.7759/cureus.4581

**Published:** 2019-05-01

**Authors:** Alexandros P Apostolopoulos, Stavros Angelis, Rahi Kiran Yallapragada, Shamsul Khan, Jila Nadjafi, Theodore Balfousias, Thiyagarajah P Selvan

**Affiliations:** 1 Orthopedics, East Surrey Hospital, Surrey and Sussex Healthcare National Health Service Trust, Redhill, GBR; 2 Orthopedics, General Hospital Hellenic Red Cross Korgialenio - Benakio, Athens, GRC; 3 Trauma and Orthopaedics, East Surrey Hospital, Surrey and Sussex Healthcare National Health Service Trust, Redhill, GBR; 4 Radiology, East Surrey Hospital, Surrey and Sussex Healthcare National Health Service Trust, Redhill, GBR; 5 Trauma and Orthopaedics, Red Cross Hospital, Athens, GRC

**Keywords:** rotator cuff, supraspinatus, magnetic resonance imaging, ultrasonography, one-stop shoulder clinic

## Abstract

Shoulder pain is a common cause of morbidity in the general population. Differential diagnosis may be difficult. Soft tissue shoulder disorders are the most common causes of shoulder pain. Noninvasive imaging techniques can reveal rotator cuff (RC) pathologies. These include ultrasonography (US) and MRI. Minimally invasive techniques such as magnetic resonance arthrography (MRA) can also be recruited when required.

We conducted a retrospective study of 61 consecutive patients with shoulder pain, who had undergone preoperative imaging in the form of US or MRI and subsequently proceeded to arthroscopic surgery. Nineteen patients had a US and 42 had an MRI preoperative imaging evaluation. This evaluation was compared to the operative findings. The US sensitivity was 87%, while specificity was 63%. The MRI accuracy rose to a sensitivity of 95% when specificity was 72%. The positive predictive value (PPV) was 64% for US and 76% for MRI. The negative predictive value (NPV) was 87% for US and 94% for MRI. The overall accuracy of the ultrasound was 73% and of the MRI 83%.

## Introduction

Shoulder pain is a common cause of morbidity in the general adult population. Prevalence of self-reported shoulder pain reaches up to 16% in the United Kingdom and rises to 26% in the elderly [1-3]. It has been suggested that only 40%-50% of people with shoulder pain will consult a general practitioner, nevertheless, it is the third most common cause of musculoskeletal consultation in primary care [3-4].

There are three major categories of pathology that can cause shoulder pain. These include soft tissue disorders, articular injury or instability, and arthritis. It has been estimated that up to 90% of lesions causing painful shoulder result from extracapsular soft tissue lesions [3]. There is a lack of consensus regarding the diagnostic criteria and the classification of shoulder disorders. This makes it difficult to estimate the frequency of the underlying causes of shoulder pain. There are reports of rotator cuff (RC) pathology prevalence in 30%-70% as a cause of shoulder pain [5-8].

It is crucial to determine the source, the extent, and the specific characteristics of the problem in the shoulder in order to be able to recommend the right treatment (conservative or surgical). History taking and clinical examination are the cornerstones of the diagnosis of shoulder disorders [3]. On the other hand, the value of history taking and clinical examination alone are limited with regard to making a decision for further management with certainty [8]. Differential diagnosis can be difficult and the most important criterion for the assessment of different imaging modalities is their ability to distinguish individual pathologies of the shoulder joint, either alone or in combination [9].

Ultrasonography (US), MRI, magnetic resonance arthrography (MRA), and arthroscopy are all used for the diagnosis of soft tissue disorders, yet their relative accuracy, cost-effectiveness, and impact on the quality of life are still uncertain [3]. Initial US results in the detection of RC tears have varied, probably due to the use of low-frequency transducers and limited experience with the examination procedure, but gradually the technique has gained its place amongst the other techniques [8,10]. MRI initially became more popular than US for preoperative diagnosis of partial and full-thickness RC tears, with high sensitivity and accuracy results. On the long run though, when considering accuracy, cost, availability, safety, and efficiency of management when used at the point of care, US is likely the best option in most settings for the diagnosis of RC tears [10]. MRA is a mildly invasive imaging technique, and use of contrast medium, gadolinium is required to be introduced in the joint [8]. Evaluations of plain X-ray and computed tomographic arthrography (CTA) are usually excluded from this kind of investigations as these techniques are recognized to have limited value in the diagnosis of soft tissue lesions [3].

The purpose of this study is to compare preoperative US and MRI accuracy for the detection of RC tears with the arthroscopy findings in our institution. This will help us draw some conclusions about the effectiveness of each method over the other.

## Materials and methods

Search strategy

Α retrospective study of all patients treated with arthroscopic surgery for shoulder pain in our clinic, by the senior author, between January 2014 and December 2017, was performed.

Inclusion criteria

The criteria for study inclusion in the retrospective study were as follows.

Population

Only patients with the presence or absence of a full-thickness or partial-thickness supraspinatus tear documented in the operative notes were included. Patients with shoulder pain resulting from other causes such as shoulder instability, arthritis, or referred pain were excluded. Patients with tears of the RC but not the supraspinatus were also excluded.

Imaging Techniques

The following diagnostic imaging techniques were included in the retrospective study:

· US

· MRI

All ultrasonograms were referred by a senior orthopedic surgeon in the one-stop Shoulder Clinic of our institute, to a radiologist experienced in the musculoskeletal US. Subsequently, the later performed and reported the US by using a high-frequency linear-array transducer.

Imaging of the shoulder with MRI was evaluated and reported by radiologists with a special interest in musculoskeletal imaging. Multi-planar imaging of the shoulder was performed using a mixture of conventional and fat-suppressed MRI and a combination of oblique coronal, oblique sagittal, and axial views (1.5 Tesla).

Evaluations of plain X-ray and CTA were excluded from the review, as these techniques are recognized to have limited value in the diagnosis of soft tissue lesions [3]. MRA imaging was also not included as in our practice, this technique is not used as a method for detecting RC tears. In our institute, MRA is usually used for suspected labral and Bankart lesions.

Eligibility Assessment

All data were assessed for inclusion by two reviewers and the senior author, and disagreements were resolved by consensus. These data included:

· documentation of supraspinatus tear in the operative notes

· imaging of supraspinatus tear during arthroscopic surgery (all surgeries were recorded on hard drives)

· reports and images of preoperative US imaging documenting supraspinatus tear

· reports and images of preoperative MRI documenting supraspinatus tear

Data analysis

The RC tendons were assessed during imaging, but only the integrity of the supraspinatus tendon was analyzed for the purpose of this study. This is the most frequently involved tendon on RC pathology. The results of US and MRI were compared separately to the operative findings. Operative findings were the reference standard for the accuracy of the US and MRI findings.

Some 95% confidence intervals (95% CI) were calculated for the accuracy of US and MRI imaging of the supraspinatus tears. The sensitivity, specificity, accuracy, positive, and negative predictive values (PPV and NPV) were also calculated for the diagnosis of this specific lesion by US and MRI. Finally, mean age, gender, and mean interval between the imaging tests and surgery were recorded.

## Results

This retrospective study comprises 104 consecutive patients with shoulder pain, who had undergone preoperative imaging in the form of US or MRI and subsequently proceeded to arthroscopic surgery. After excluding patients not meeting the sample's criteria, 61 patients remained in the study group.

**Figure 1:**
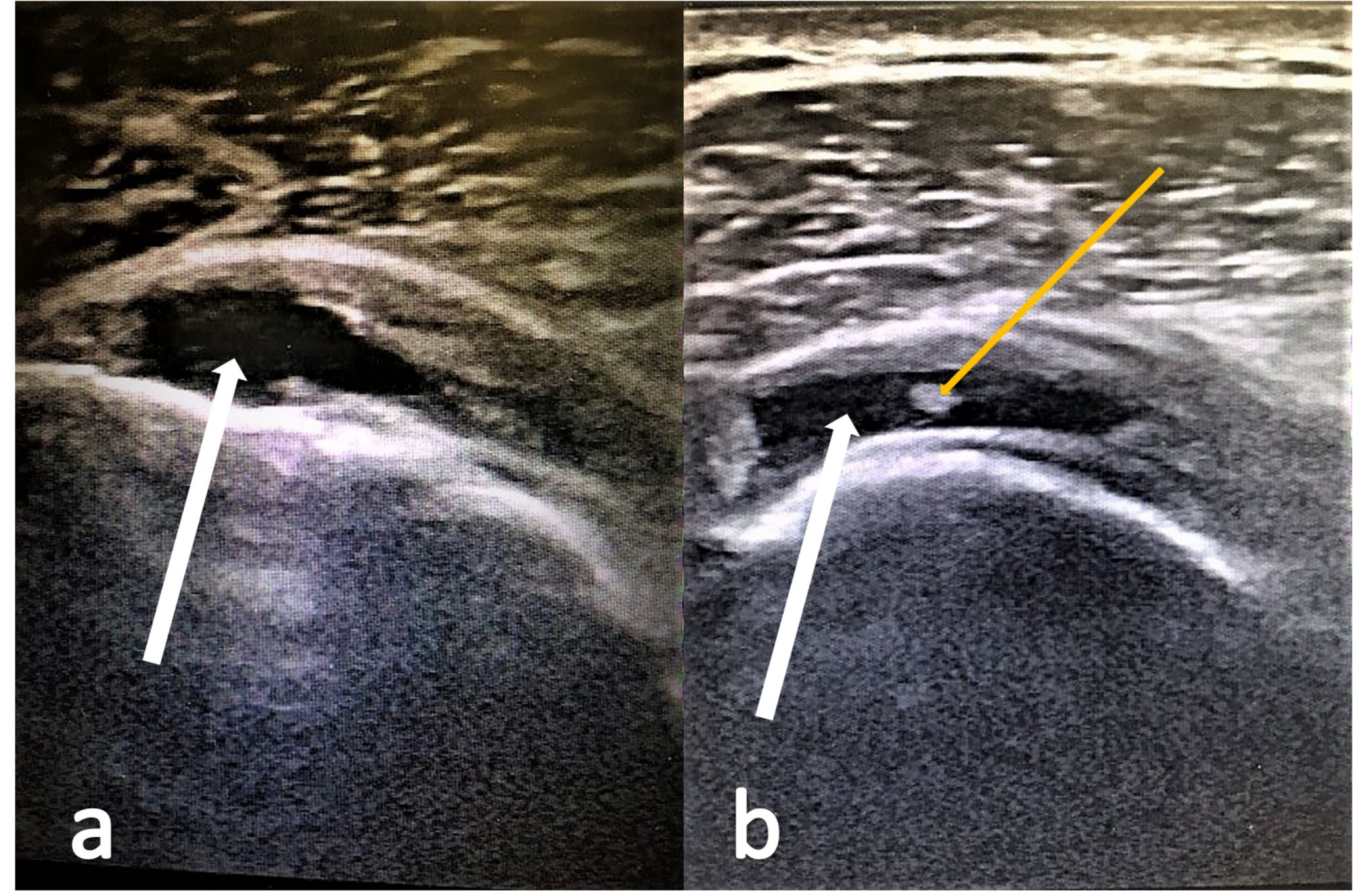
Supraspinatus tears depicted in US. a. The white arrow points to a full-thickness supraspinatus tear. b. The white arrow points to a full-thickness supraspinatus tear. The yellow arrow points to an intra-tendinous inflammation of the supraspinatus tendon. US, ultrasonography.

Preoperative US was performed on 19 patients (10 males and 9 females, mean age 55.52 years old, mean interval between US and surgery 23 days). Ultrasonography correctly diagnosed seven out of eight tears (sensitivity of 87.5%) (Figure 1). There were seven true-negative and four false-positive ultrasounds (specificity of 63.6%). Arthroscopy on the four patients with false-positive US revealed one biceps tear and three RC tears, but no supraspinatus tear. All features of US accuracy are demonstrated in Tables 1-2.

**Table 1:** US findings correlated to arthroscopic findings. US, ultrasonography; TP, true positive; FN, false negative; FP, false positive; TN, true negative.

US	Arthroscopy+	Arthroscopy-
US +	7 (TP)	4 (FP)
US -	1 (FN)	7 (TN)

**Table 2:** US results. US, ultrasonography; pts, patients; PPV, positive predictive value; NPV, negative predictive value.

US Results No : 19 pts	
PPV	63.6%
NPV	87.5%
Sensitivity	87.5%
Specificity	63.6%
Accuracy	73%

**Figure 2:**
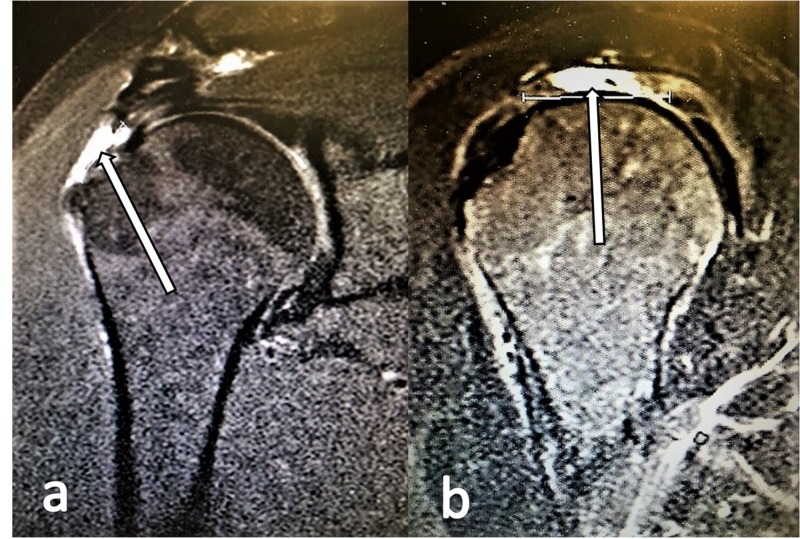
Supraspinatus tears depicted in MRI scans. a. T2-weighted oblique coronal MRI view with a high-intensity signal (white arrow) revealing a full-thickness tear of the supraspinatus. b. T2-weighted oblique sagittal MRI view with a high-intensity signal (white arrow) revealing a full-thickness tear of the supraspinatus.

Preoperative MRI was performed on 42 patients (20 males and 22 females, mean age 56.71 years old, mean interval between US and surgery 1.4 months). MRI accurately identified 19 of the 20 tears (sensitivity of 95%) (Figure 2). There were 16 true-negative and 6 false-positive tears on MRI (specificity of 72.7%). The false-positive results proved to be three partial articular supraspinatus tendon avulsion (PASTA) lesions, two Bankart lesions, and one partial thickness tear located in the infraspinatus tendon. All features of MRI accuracy are demonstrated in Tables 3-4.

**Table 3:** MRI findings correlated to arthroscopic findings. TP, true positive; FN, false negative; FP, false positive; TN, true negative.

MRI	Arthroscopy+	Arthroscopy-
MRI +	19 (TP)	6 (FP)
MRI -	1 (FN)	16 (TN)

**Table 4:** MRI results. pts, patients; PPV, positive predictive value; NPV, negative predictive value.

MRI Results No : 42 pts	
PPV	76%
NPV	94.1%
Sensitivity	95%
Specificity	72.3%
Accuracy	83.3%

## Discussion

Shoulder pain is a common cause of morbidity in the general adult population and also for musculoskeletal consultation in primary care. Pain is usually poorly localized, with the exception of pain occurring in the acromioclavicular joint and, therefore, differential diagnosis may be difficult. Even today, history taking and clinical examination are the cornerstones of the diagnosis of shoulder disorders. Specifically, for extracapsular soft tissue lesions, the majority of studies evaluate the ability of clinical examination to identify patients with RC tears. A meta-analysis and systematic review performed to evaluate the effectiveness of diagnostic tests for the assessment of shoulder pain due to soft tissue disorders, for the National Health Service (NHS) Research and Development Health Technology Assessment (HTA) Programme in the United Kingdom, suggests that clinical examination as a whole, when carried out by relatively specialized clinicians such as orthopedics, may be useful at ruling out RC tears but less accurate at detecting such tears when they are present. Insufficient evidence was found to recommend any specific clinical examination test or set of tests or to provide an indication of the accuracy of clinical examination at differentiating RC disorders from other causes of shoulder pain [3].

What clinical examination and history taking cannot give a definite answer to, is whether to recommend conservative or surgical treatment. It is easily assumed that alone these tests are limited with regard to making a decision for further management with certainty [8]. Therefore, recruitment of imaging techniques is imperative.

For many years, arthrography was the only technique available for the detection and imaging of RC tears. It was invasive with well-described complications and risks [11-12]. Arthrography has gradually been replaced by US, MRI, and MRA. Even CTA and X-rays can give direct or indirect information about RC lesions. MRA is a mildly invasive imaging technique that is gradually gaining its place among the most popular imaging techniques for the depiction of RC tears. MRA may have some role in the diagnosis of full-thickness and possibly partial thickness tears, but any such benefit must be set against the invasiveness and potential discomfort to patients from the procedure [3]. CTA and X-rays are known to have limited value in the diagnosis of soft tissue lesions. This study evaluates the accuracy of the more commonly used US and MRI for RC tears.

Seltzer et al. were the first to report US of the shoulder for the detection of fluid and intra-articular loose bodies within the joint [13]. Their paper is not as old as one might assume as it was published in 1980, but even the pioneers in this field could not have predicted the progress achieved. US has proved its role in assessing tendons of the RC and a high frequency (7-15 MHz) linear-array probe is required for satisfactory images [3, 8-9, 14]. US of the shoulder is utilized in secondary, tertiary and, increasingly, primary healthcare settings to evaluate the integrity of the RC [15]. It consists of a portable, noninvasive examination that has practically no adverse effects and is well tolerated by the patient. It allows dynamic visualization of the tendons during movement of the shoulder and interaction with the patient; it is cost effective and time efficient [8, 16]. To the time and cost effectiveness, one can also add the fact that even orthopedic surgeons may be trained to acquire the skill and perform US in the clinic at the first point of contact [16]. This saves an enormous amount of time and money and reduces the workload and financial burden on the Radiology Department. Moreover, it reduces the waiting time of the patients as they can be fully clinically and radiologically assessed in a one-stop clinic. In our study, this is verified, as the mean interval between the ultrasound and surgery was only 23 days when the mean interval between MRI and surgery was 1.4 months. However, operator dependence and a long-learning curve are frequently considered to be US's limitation. US also seems to generate low-quality depiction of the RC in patients who are obese, muscular, or have severely restricted shoulder movement [17-18].

In 1986, Kneeland et al. for the first time reported their results in the use of MRI for detecting shoulder RC tears [19]. The authors predicted that this procedure will not replace sonography as it is much more expensive. They mentioned though, that the technique will be useful in cases where US yields indeterminate results, in institutions where no one is trained to perform US of the shoulder, and in cases where size and location of the tear need to be precisely depicted. Since then, this technique has been widely used in secondary and tertiary healthcare practice. MRI is a noninvasive method of imaging that is unique in allowing high-resolution images in multiple planes [15]. Most orthopedic surgeons are trained to recognize the appearance of a full-thickness tear as a high-intensity signal on a T2-weighted image that extends from the articular surface of the RC to the subacromial or subdeltoid bursa. Localizing a small partial-thickness tear to the RC crescent may be helpful for the shoulder surgeon, who may then decide to only debride, but not repair, the cuff defect [20]. If a full-thickness tear is observed, it is important to document whether or not the entire anterior-to-posterior width of the supraspinatus tendon is involved. In RC tears that involve the entire tendon, the tendon edge can retract medially, where it becomes extremely difficult to grasp and to reattach to the greater tuberosity [20]. Moreover, MRI can give information with regard to the quality of the tendon (muscle atrophy and fatty degeneration), or retraction of the tendon, and can, therefore, be a significant tool for preoperative planning. The strength of the magnet, the sequences used in the examinations, and the person (e.g. consultant radiologist, musculoskeletal radiologist, trainee, orthopedic surgeon) interpreting and reporting the test may all affect the results [15]. MRI has some absolute contraindications, such as the presence of intracerebral aneurysm clips, cardiac pacemakers, automatic defibrillators, biostimulators, implanted infusion devices, cochlear implants, and metallic orbital foreign bodies [20-21]. It is also expensive and time-consuming. 

This study was designed to evaluate US and MRI for the diagnosis of RC tears in terms of accuracy, cost and time-effectiveness, availability, safety and efficiency of management when used in our institution. The limitation of this study is that the patients had undergone either US or MRI as part of their preoperative assessment and not both. 

In the US group, (N = 19) eight had full thickness RC tears, seven were diagnosed accurately, while one was misdiagnosed with no tear. Out of 11 intact supraspinatus tendons seven were diagnosed accurately, two were diagnosed as partial-thickness tears, and two as full-thickness tears of the supraspinatus. In the MRI group, (N = 42) 19 of the 20 supraspinatus tendon tears were accurately diagnosed. Out of 22 intact supraspinatus tendons, 16 were correctly diagnosed, and six were diagnosed as RC tears of the supraspinatus. The sensitivity for the US group was 87% and for the MRI group was 95%. The specificity was 63% and 72%, respectively. The NPV was 87% for US and 94% for MRI. The overall accuracy of the ultrasound was 73% and of the MRI 83%. The cost of the shoulder ultrasound scan in our trust is 34 £ and the relevant cost of the MRI is 134 £. Moreover, the US was performed in our one-stop Shoulder Clinic. In that way, there was a significant cost reduction by saving 100 £ per patient in the imaging studies. There was also an important saving in time and money by reducing the number of follow-ups that would have been required if the patient was referred for an MRI scan.

In our practice, we request MRI scans if there is a clinical suspicion of a superior labral tear from anterior to posterior (SLAP) or a Bankart lesion. We also proceed to MRI investigations in case of chronic full-thickness tears in order to look for tendon retraction and muscle atrophy and perform our preoperative planning. The results of our study are comparable with the results that Roy et al. have published in a meta-analysis that the authors performed by reviewing 264 articles [10]. To our knowledge, this is the most recent meta-analysis that investigates the accuracy of imaging techniques in shoulder tendinopathy. The authors conclude that the diagnostic accuracy of US, MRI, and MRA in the characterization of full-thickness RC tears was high with overall estimates of sensitivity and specificity over 90%. As for partial RC tears and tendinopathy, overall estimates of specificity were also high (>90%), while sensitivity was lower (67%-83%).

## Conclusions

Tears of the supraspinatus can be identified using ultrasound and MRI with comparable accuracy. US being a dynamic study and better tolerated by the patient, can, therefore, be used as the first-line investigation for RC tear to reduce the waiting time and cost of investigation, where appropriate skills are available (trained operators). US is much cheaper (34 £ in our trust), compared to the MRI scan (134 £ in our trust). However, in clinical situations where other shoulder conditions such as articular cartilage injuries or labral tears are suspected (e.g., in cases where glenohumeral instability in younger patients or osteoarthritis in older patients overlap with RC disorders), an MRI or an MRA should be used.
